# Evaluation of the SARS-CoV-2 positivity ratio and upper respiratory tract viral load among asymptomatic individuals screened before hospitalization or surgery in Flanders, Belgium

**DOI:** 10.1371/journal.pone.0259908

**Published:** 2021-11-11

**Authors:** Laura Heireman, Steven Abrams, Peggy Bruynseels, Reinoud Cartuyvels, Lize Cuypers, Pieter De Schouwer, Wim Laffut, Katrien Lagrou, Niel Hens, Erwin Ho, Elizaveta Padalko, Marijke Reynders, Sarah Vandamme, Nathalie Van der Moeren, Walter Verstrepen, Philippe Willems, Reinout Naesens

**Affiliations:** 1 Department of Laboratory Medicine, ZiekenhuisNetwerk Antwerpen, Antwerp, Belgium; 2 Data Science Institute, Interuniversity Institute for Biostatistics and statistical Bioinformatics (I-BioStat), UHasselt, Hasselt, Belgium; 3 Global Health Institute, Family Medicine and Population Health, University of Antwerp, Antwerp, Belgium; 4 Department of Laboratory Medicine, Jessa Hospital, Hasselt, Belgium; 5 Department of Laboratory Medicine and National Reference Center for Respiratory Pathogens, University Hospitals Leuven, Leuven, Belgium; 6 Department of Laboratory Medicine, Heilig Hart Hospital, Lier, Belgium; 7 Centre for Health Economic Research and Modelling Infectious Diseases, University of Antwerp, Antwerp, Belgium; 8 Department of Laboratory Medicine, Sint-Maarten Hospital, Mechelen, Belgium; 9 Department of Laboratory Medicine, University Hospital Ghent, Ghent, Belgium; 10 Department of Laboratory Medicine, Sint-Jan Hospital, Bruges, Belgium; 11 Department of Laboratory Medicine, Antwerp University Hospital, Antwerp, Belgium; 12 Department of Laboratory Medicine GasthuisZusters Antwerpen, Antwerp, Belgium; University of Hong Kong, HONG KONG

## Abstract

**Introduction:**

The incidence of Severe Acute Respiratory Syndrome Coronavirus 2 (SARS-CoV-2) infections in the Belgian community is mainly estimated based on test results of patients with coronavirus disease (COVID-19)-like symptoms. The aim of this study was to investigate the evolution of the SARS-CoV-2 reverse transcriptase polymerase chain reaction (RT-PCR) positivity ratio and distribution of viral loads within a cohort of asymptomatic patients screened prior hospitalization or surgery, stratified by age category.

**Materials/Methods:**

We retrospectively studied data on SARS-CoV-2 real-time RT-PCR detection in respiratory tract samples of asymptomatic patients screened pre-hospitalization or pre-surgery in nine Belgian hospitals located in Flanders over a 12-month period (1 April 2020–31 March 2021).

**Results:**

In total, 255925 SARS-CoV-2 RT-PCR test results and 2421 positive results for which a viral load was reported, were included in this study. An unweighted overall SARS-CoV-2 real-time RT-PCR positivity ratio of 1.27% was observed with strong spatiotemporal differences. SARS-CoV-2 circulated predominantly in 80+ year old individuals across all time periods except between the first and second COVID-19 wave and in 20–30 year old individuals before the second COVID-19 wave. In contrast to the first wave, a significantly higher positivity ratio was observed for the 20–40 age group in addition to the 80+ age group compared to the other age groups during the second wave. The median viral load follows a similar temporal evolution as the positivity rate with an increase ahead of the second wave and highest viral loads observed for 80+ year old individuals.

**Conclusion:**

There was a high SARS-CoV-2 circulation among asymptomatic patients with a predominance and highest viral loads observed in the elderly. Moreover, ahead of the second COVID-19 wave an increase in median viral load was noted with the highest overall positivity ratio observed in 20–30 year old individuals, indicating they could have been the hidden drivers of this wave.

## Introduction

The incidence of severe acute respiratory syndrome coronavirus 2 (SARS-CoV-2) infections in the Belgian community is currently estimated based on real-time reverse transcriptase polymerase chain reaction (RT-PCR) and rapid antigen test results of patients with coronavirus disease (COVID-19)-like symptoms and, to a lesser extent, of individuals with epidemiological arguments for testing. Large studies reported however that 1–13% of SARS-CoV-2 infected individuals remain asymptomatic with smaller studies reporting even higher proportions (up to 88%) [1]. Real-time RT-PCR is considered the reference standard for SARS-CoV-2 detection with lower cycle threshold (Ct) values corresponding to higher viral RNA concentrations and higher infectiousness [2]. Some studies reported lower SARS-CoV-2 viral loads in the nasopharynx/oropharynx of asymptomatic patients (children and adults) with shorter viral shedding compared to symptomatic patients [3–7]. Other studies observed similar viral loads in respiratory tract samples from asymptomatic or pre-symptomatic persons compared to symptomatic patients and long-term virus shedding in asymptomatic patients [2,8–10]. In addition, the isolation of cultivable virus from persons with asymptomatic SARS-CoV-2 infection has been described [2,8]. Based on the observed dynamics of SARS-CoV-2 viral loads in upper respiratory tract specimen and the incubation period for COVID-19, a substantial proportion of pre-symptomatic transmission of SARS-CoV-2 is probable [2,11–13]. The asymptomatic and pre-symptomatic cases may represent an important source of potentially transmissible virus whose early identification may help in controlling the community spread of SARS-CoV-2 [1,2,8,14,15].

In addition to the lack of inclusion of asymptomatic cases in the current surveillance data, the proportion of individuals who do have symptoms or epidemiological risk factors that are being tested is estimated to be far below 100% [16–18]. More specifically, overall underreporting factors (total SARS-CoV-2 infections vs. confirmed COVID-19 cases) of 33 (95% credible interval (CrI): 32.0–34.7, on March 22, 2020), 9 (on 12 April 2020) and 3.5 (on 7 June 2020) have been estimated for the early phase of the epidemic in Belgium based on a stochastic compartmental modeling approach [19]. Surveillance of SARS-CoV-2 community spread based on these test results leads subsequently to a substantial underestimation of the true population incidence and a delayed image of the epidemiological situation.

Accurate and up-to-date knowledge of the true size of the epidemic is however essential for timely and efficient implementation of measures to control SARS-CoV-2 community spread [15,20]. To date, no data are available concerning asymptomatic SARS-CoV-2 carriership and viral loads in the general Belgian population nor at the regional level. Furthermore, the role of the different age categories in the viral spread has not been fully elucidated [21].

The aim of this study was to investigate the evolution of the SARS-CoV-2 (RT)-PCR positivity ratio and distribution of viral loads within a cohort of asymptomatic patients screened prior hospitalization or surgery, stratified by age category.

## Materials and methods

We retrospectively studied data on real-time RT-PCR detection of SARS-CoV-2 in respiratory tract samples of asymptomatic patients collected in nine Belgian hospitals located in Flanders over a 12-month period (from 1 April 2020 to 31 March 2021). The hospitals ZiekenhuisNetwerk Antwerpen (ZNA, Antwerp), GemeenschapsZusters Antwerpen (GZA, Antwerp), Antwerp University Hospital (UZA, Antwerp), Heilig Hart Hospital (Lier), Sint-Maarten Hospital (Mechelen), Ghent University Hospital (Ghent), University Hospitals Leuven (Leuven), Jessa Hospital (Hasselt) and Sint-Jan Hospital (Bruges) participated in this study, representing the five Flemish provinces. During the study period, all patients requiring hospitalization for indications other than COVID-19 and all patients requiring a surgical procedure in one of the nine participating hospitals were screened with SARS-CoV-2 real-time RT-PCR on a nasopharyngeal or combined oropharyngeal/nasal specimen as part of the standard hospital infection control protocol. Screenings of high/low risk contacts or individuals with symptoms were excluded, as well as non-risk related screenings when they could not be distinguished from risk-related data (for example, data from Sint-Jan Hospital in the period December and January were excluded since extensive hospital staff screenings were also included and not excludable from the data set).

The patient information from each hospital is summarized in Table 1 together with the viral RNA extraction procedures and the SARS-CoV-2 real-time RT-PCR assays performed in each of the included hospitals. In addition to the real-time RT-PCR result, the parameters postal code, age and RT-PCR Ct-value (for positive results if available) were retrieved from the laboratory information system of each hospital. The conversion of Ct-values to viral loads was performed taking into account a conversion factor based on the measurement of standards on each RT-PCR system in each participating hospital. A viral load of ≥5log RNA copies/mL was defined as strongly positive.

**Table 1:** Overview of included number of test results, applied real-time SARS-CoV-2 RT-PCR methods and targeted genes per hospital.

Hospital	Province	Postal code	Included number of test results	Applied SARS-CoV-2 RT-PCR methods	Targeted genes
ZiekenhuisNetwerk Antwerpen	Antwerp	2020, 2660, 2170, 2980,	6338	Roche MagNa Pure 96 (extraction) + LightCycler 480 (amplification)	N gene
				Thermo Fisher KingFisher Flex (extraction) + Quantstudio 5 (amplification)	N gene, S gene and ORF1ab gene
				Roche Cobas 6800	ORF1ab gene, E gene
Gemeenschaps-Zusters Antwerpen	Antwerp	2610, 2018	41431	Promega Maxwell + Thermo Fisher Quantstudio 7 flex qPCR cycler® (amplification)	N1 gene
				Seegene STARlet (extraction) + Thermo Fisher Quantstudio 7 flex qPCR cycler® (amplification)	
Antwerp University Hospital	Antwerp	2650	26708	GeneXpert Xpress SARS-CoV-2	E and N2 gene
				BioMérieux NucliSens EasyMag® or Qiagen QiaSymphony® (extraction) + Roche Cobas LightCycler 480 II® or Cobas z480 (amplification)	E gene
				BD MAX® platform	N1 and N2 gene
Ghent University Hospital	East Flanders	9000	12016	GeneXpert Xpress SARS-CoV-2	E and N2 gene
				Hologic Panther Fusion® SARS-CoV-2	ORF1ab gene
				NUCLISENS easyMAG (extraction) + CFX96 (amplification)	E-gene
				ThermoFisher KingFisher Flex (extraction) + QuantStudio 5 (amplification)	N gene, S gene and ORF1ab gene
University Hospitals Leuven	Flemish Brabant	3000	66204	GeneXpert Xpress SARS-CoV-2	E and N2 gene
				Hologic Panther Fusion® and Aptima SARS-CoV-2	ORF1ab gene
				Abbott Alinity m SARS-CoV-2	RdRp and N gene
				ThermoFisher KingFisher Flex or NUCLISENS easyMAG (extraction) + Thermo Fisher QuantStudio Dx (amplification) (in-house kit)	E gene
				Thermo Fisher KingFisher Flex (extraction) + Thermo Fisher QuantStudio 7 Flex (amplification) (TaqPath COVID-19 kit)	N, S and ORF1ab gene
Jessa General Hospital	Limburg	3500	28724	Abbott Alinity m SARS-CoV-2	RdRp and N gene
				Abbott m2000 sp (extraction) + Thermo Fisher Quantstudio 7 (amplification)	N-gene
Heilig Hart Hospital	Antwerp	2500	7350	GeneXpert Xpress SARS-CoV-2	N2 and E-gene
				ELITe InGenius®	E-gene
				Seegene Allplex™ SARS-CoV-2	N gene, E gene and RdRP/S gene
				Luminex ARIES® SARS-CoV-2	E gene, N gene and ORF1ab gene
General Hospital Sint-Maarten	Antwerp	2800	12246	ELITe InGenius®	E, N and RdRP gene
				Thermo Fisher KingFisher Flex (extraction) + Thermo Fisher QuantStudio (amplification)	N gene, S gene and ORF1ab gene
				Luminex ARIES® SARS-CoV-2	N gene and ORF1ab gene
General Hospital Sint-Jan	West Flanders	8000	54908	Qiagen QiaSymphony or Roche Cobas 4800 (extraction) + Thermo Fisher Viia-7 (amplification)	N2 and RdRP gene
				Roche Cobas 6800	ORF1- and E-gene

The evolution of the overall SARS-CoV-2 positivity ratio and median viral load over time was visually represented per month. In order to put these numbers in perspective, we have added the number of new hospitalizations and positivity ratio in the general Flemish population over time for the provinces of Limburg, Antwerp, East Flanders, West Flanders and Flemish Brabant combined, as reported online by the Belgian Scientific Institute of Public Health, Sciensano [22]. Also the Oxford stringency index together with the time at which important mitigation measures were put into place are showed [23,24].

Furthermore, positivity ratios were compared between different provinces and age groups (ten-year age groups, except for the oldest age category including patients of 90 years and above, denoted by 90+) and the distributions of viral loads in these provinces and age groups were visualized.

A comparison of proportions was performed using a Chi-square test (or Fisher’s exact test in case of small cell counts) whereas the median (log10-transformed; hereafter referred to as log-transformed) viral loads across age groups and provinces were compared using a non-parametric Kruskal-Wallis rank sum test with pairwise comparisons based on the Wilcoxon rank sum test (and Bonferroni-Holm (BH) p-value correction for multiple testing). In order to describe the trends in the observed SARS-CoV-2 positivity data, we used a flexible generalized additive model (GAM) including age (in years), time (in days) and province (Limburg, Antwerp, East Flanders, West Flanders and Flemish Brabant) as covariates [25]. More specifically, we used a binomial GAM with logit- or complementary log-log link function to describe the nonlinear evolution of the positivity ratio and we compared different models based on the Akaike Information Criterion (AIC). Next to (smooth functions of the continuous) covariates, we considered two- and three-way interaction effects in the model building procedure. The final model results are presented graphically in the results section. Similarly, we use a GAM with underlying normal distribution (and identity link) to model the evolution of log-transformed viral loads over time.

Statistical significance was defined based on a two-sided *p*-value smaller than 0.05. Statistical analyses were performed using the free statistical software program R [26]. Data from all centers were fully anonymized before processing. Ethical approval was obtained by the local Ethics Committees of the participating hospitals (ZNA n° 5416, Antwerp University Hospital n° 20/36/462, GemeenschapsZusters Antwerpen n° 200906RETRO, University Hospitals Leuven N° S64903, General Hospital Sint-Jan n° 2864, Jessa General Hospital n° f/2020/176, Heilig Hart Hospital n° 2021.06, General Hospital Sint-Maarten n° EC2127).

## Results

In total, 255925 SARS-CoV-2 real-time RT-PCR test results, obtained from patients domiciled in the Flemish provinces Antwerp (99030 test results of which 1082 (1.09%) were positive), East Flanders (18653; 197 (1.06%) positive), West Flanders (54783; 1331 (2.43%) positive), Limburg (36049; 296 (0.82%) positive) and Flemish Brabant (Leuven Arrondissement; 47410; 340 (0.72%) positive) (i.e., postal codes 2000–2999, 3000–3999, 8000–8999 and 9000–9999), were included in this study. Overall, we observe large differences in positivity ratio across the different Flemish provinces. The number of tests performed by each hospital are, in decreasing order, 66204 (University Hospitals Leuven), 54908 (Sint-Jan Hospital), 41431 (Gemeenschaps-Zusters Antwerpen), 28724 (Jessa Hospital), 26708 (Antwerp University Hospital), 12246 (Sint-Maarten Hospital), 12016 (Ghent University Hospital), 7350 (Heilig Hart Hospital) and 6338 (ZiekenhuisNetwerk Antwerpen). In the remainder of the manuscript, we will refer to province as the reported province of residence of each of the individual patients.

Furthermore, 2421 positive test results for which a viral load measurement was reported were considered for further analysis. The median age of tested patients was 54 years (first and third quartile 33 and 69 years, respectively). We included the following number of patients from each age category: 5% (N = 12067) [0,10) years, 6% (N = 14503) [10,20) years, 10% (N = 25246) [20,30) years, 13% (N = 32584) [30,40) years, 11% (N = 28732) [40,50) years, 15% (N = 38946) [50,60) years, 16% (N = 41423) [60,70) years, 14% (36667) [70,80) years, 8% (N = 21186) [80,90) years and 2% (N = 4571) 90+ years. An unweighted overall SARS-CoV-2 real-time RT-PCR positivity ratio of 1.27% (3246/255925) among all a-/pre-symptomatic patients who were screened pre-hospitalization or pre-surgery was observed.

**Fig 1:**
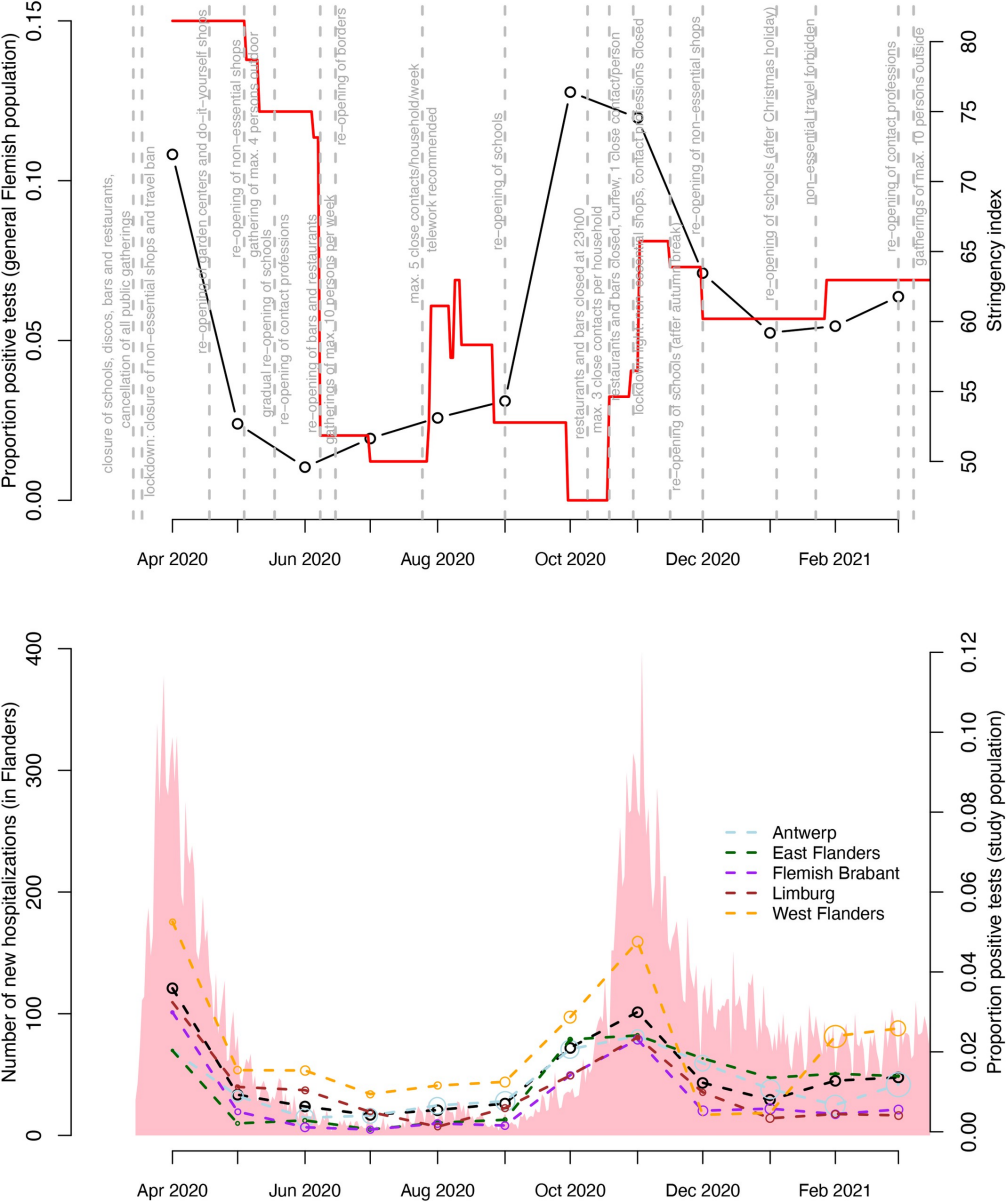
**Evolution of the monthly SARS-CoV-2 real-time RT-PCR positivity ratio in the Flemish population (black solid line) and in a-/pre-symptomatic patients screened pre-hospitalization/pre-surgery by province and overall (black dots and dashed line) (in Flanders) together with the daily number of SARS-CoV-2 hospitalizations in Flanders (pink shaded area) as a function of time (from 1 April 2020 to 31 March 2021).** The size of the dots is proportional to the number of observations that are available. The Oxford stringency index (red solid line) is presented together with the time at which important mitigation measures (gray) were put into place.

In Fig 1, we graphically depict the evolution of the SARS-CoV-2 positivity ratio per month in our study population (overall (black dots and dashed line) and by province) and the general Flemish population (Sciensano data) between 1 April 2020 and 31 March 2021. Also the observed daily number of new hospitalizations in Flanders (pink shaded area) and the Belgian Oxford stringency index together with the time at which important mitigation measures (gray) were put into place are displayed within this time period. From the graph, it is clear that in spite of regional differences (i.e., between provinces) the evolution of the positivity ratio in our study population resembles the evolution in hospitalizations. A similar temporal evolution is observed between the positivity ratio in our study population and the one in the general Flemish population, except that the SARS-CoV-2 peak occurred earlier for the latter (October instead of November 2020). Before the second wave in October 2020, the stringency index was 47–53 (September to beginning of October) with the most important relaxation of stringent measures being the reopening of the schools at the first of September 2020. Based on the unweighted overall positivity ratio (black dots), the highest peak % SARS-CoV-2 carriership was observed during the first and second Belgian COVID-19 wave (i.e., 3.59% in April and 3.00% in November 2020).

Regional differences in SARS-CoV-2 positivity ratio are also shown in Fig 1 with the highest percentages of positive tests observed in West Flanders (orange points) during the first COVID-19 wave and second COVID-19 wave. There is a large percentage of positive tests observed in West Flanders in February 2021 (possibly already to be observed from December given the omission of part of the data from Sint-Jan Hospital), which is different from the SARS-CoV-2 positivity ratios in the other provinces.

A more granular look at the evolution of the SARS-CoV-2 positivity ratio over time in terms of bi-weekly percentages and by province can be found in S1 Fig.

**Fig 2:**
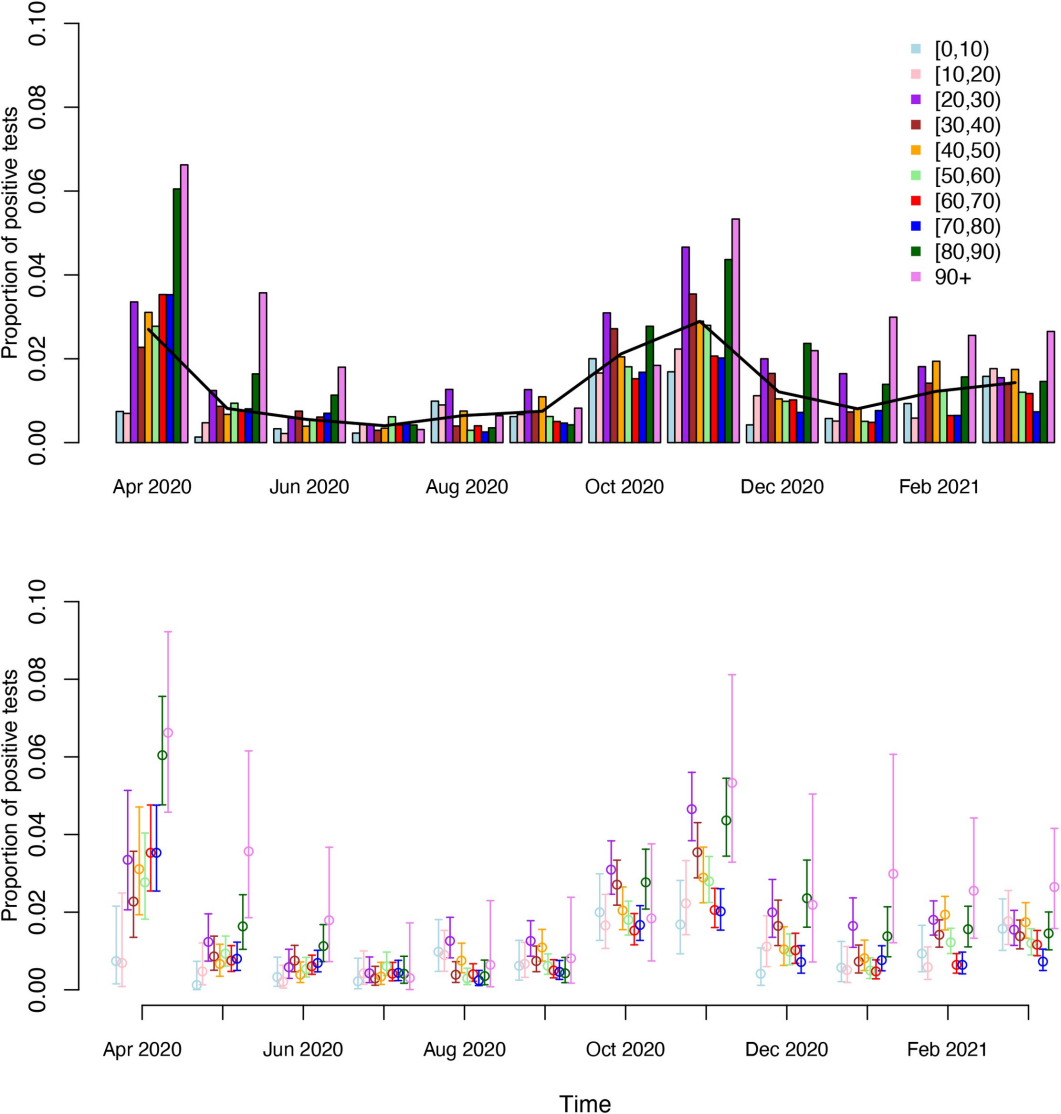
Evolution of the monthly SARS-CoV-2 real-time RT-PCR positivity ratio in a-/pre-symptomatic patients screened pre-hospitalization/pre-surgery by ten-year age group as a function of time (from 1 April 2020 to 31 March 2021). The black solid line shows the age-weighted overall SARS-CoV-2 positivity ratio based on the age distribution in Belgium anno 2020. Error bars represent 95% exact Clopper-Pearson confidence limits for the age-specific positivity ratios.

The highest overall positivity ratio is observed in the age groups 90+ (2.8%), [80,90) (1.8%) and [20,30) (1.9%) (0.9–1.4% for the other age groups). Age-specific SARS-CoV-2 carriership over time is graphically depicted in Fig 2. More specifically, we show the monthly evolution of the percentage of SARS-CoV-2 positive patients that were screened pre-hospitalization or pre-surgery and without COVID-19 symptoms by ten-year age group. Combining data from the entire time period, there exists a significant difference in positivity ratio across different age groups (Chi-square p-value < 0.0001). A comparison of the monthly positivity ratios among age groups indicates significant differences in each month, except for June and July in which no significant pairwise differences were observed. From the figure, one can clearly observe that the highest percentage of positive tests is observed in the [80, 90) and 90+ age groups over the entire time period (except for the summer period including July, August and September). SARS-CoV-2 positivity ratios were significantly different in the 80+ year old across all time periods, except for the abovementioned summer months, as compared to their younger counterparts based on pairwise Chi-square tests. More specifically, during April 2020 a significantly different positivity ratio was observed in each of the age groups [80,90) and 90+ compared with the age groups [0,10), [10,20), [30,40) and [50,60) (Fisher’s exact BH adjusted p-values < 0.036) with higher positivity ratios for the elderly. Moreover, in May, the positivity ratio in all age groups, except for [20,30) and [80,90), was found to be significantly different from the positivity ratio in the highest age group. In August, prior to the onset of the second COVID-19 wave, the positivity ratio was estimated to be highest in the youngest age groups with a significant difference in positivity ratio as compared to the one in older age groups between 30 and 90 years of age. Moreover, the virus circulated predominantly in the age group [20,30) before the second wave emerged (August—September 2021). The positivity ratio increased in all age groups between September and October 2020. During October 2020, the positivity ratio was found to be highest in the age groups [20,30] and [30,40), being significantly different from positivity ratios in the age groups [50,60), [60,70) and [70,80). The SARS-CoV-2 carriership was found to be highest in the [20,30) and [30,40) age groups, together with the oldest age groups (80+), and significantly different from the positivity ratios in other age groups during November and December as well.

After December 2020, only in February 2021 significant differences in positivity ratio were detected with significantly different values in age groups [20,30), [30,40), [40,50), and 80+ on the one hand and all other age categories on the other hand. The SARS-CoV-2 positivity ratio is not significantly different between wave 1 (i.e. April 2020) and wave 2 (i.e. October—November 2020) for all age groups, except for [60,70) (Fisher’s exact BH corrected p-value = 0.001), [70,80) (p-value = 0.003) and [80,90) (p-value = 0.002). Throughout the course of the epidemic, SARS-CoV-2 positivity was found to be smallest in the youngest age groups (i.e., [0,10), [10,20)), albeit that these percentages were considerably higher in August 2020 and March 2021.

**Fig 3:**
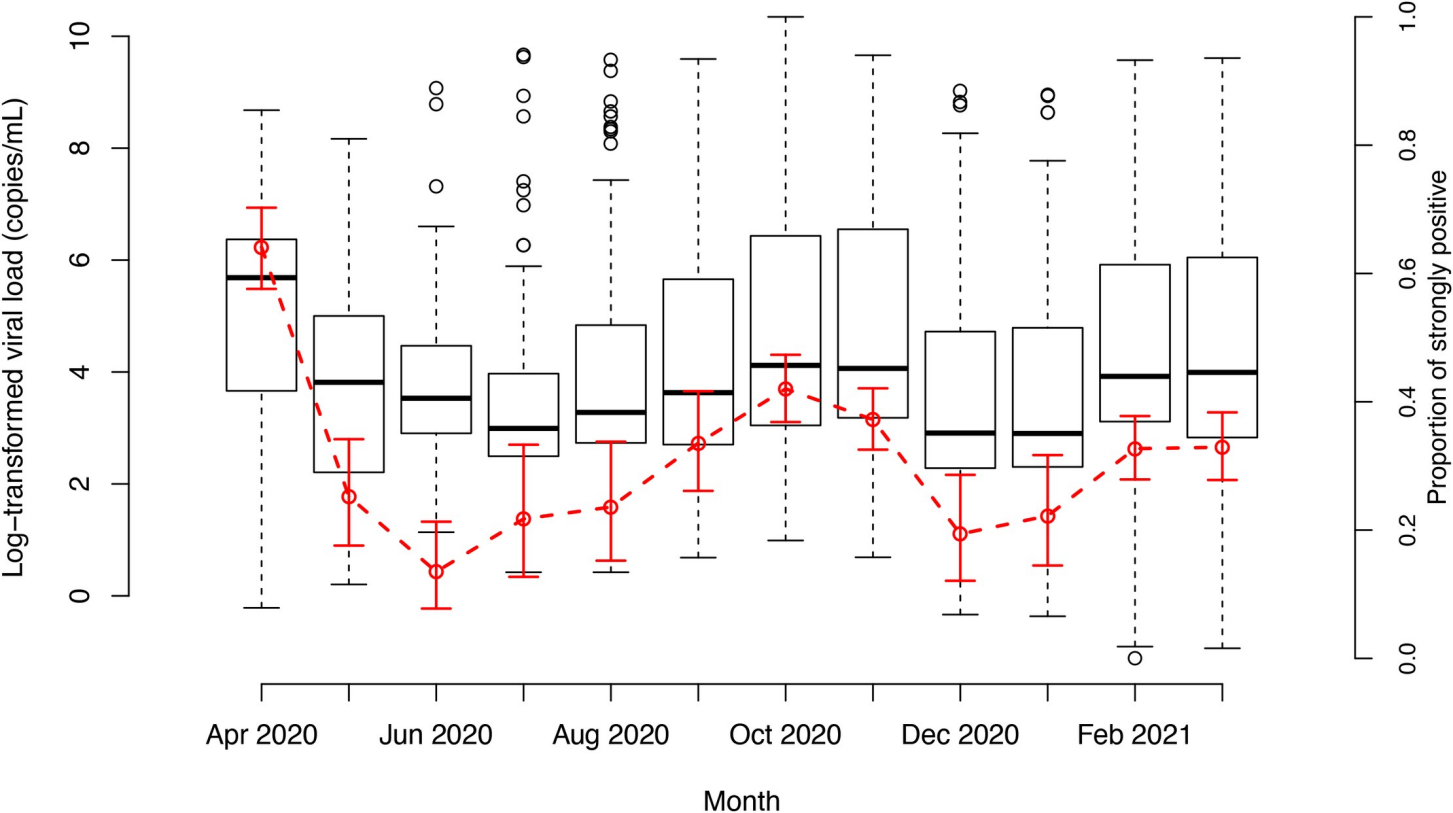
Boxplots showing the evolution of the (distribution of the) log-transformed viral load (in copies/mL) for SARS-CoV-2 positive a-/pre-symptomatic patients by month (from 1 April 2020 to 31 March 2021) and the evolution of the percentage of strongly positive patients among the SARS-CoV-2 positive a-/pre-symptomatic patients by month (red dots) with pointwise 95% exact Clopper-Pearson confidence intervals.

The general evolution of the (log-transformed) viral load over time (by month) as well as the evolution of the proportion of strongly positive patients among all PCR positive patients (i.e., ≥5log RNA copies/mL) is illustrated in Fig 3. The median viral load follows a similar temporal evolution as was seen in the positivity rate. Overall, the median log-transformed viral loads were significantly different over time (Kruskal-Wallis two-sided p-value < 0.0001). More specifically, median values were highest in April 2020 and significantly different from median viral loads in all other months, except for those observed in October and November 2020. Similar median log-transformed viral loads were observed between May and September 2020 on the one hand and December 2020 to January 2021 on the other hand. Clearly, the median log-transformed viral load decreased from April 2020 (first wave) until July 2020 after which median values increased until the end of the second wave (November 2020). The trend in proportion of strongly positive patients is very similar to the evolution in median viral load by month.

**Fig 4:**
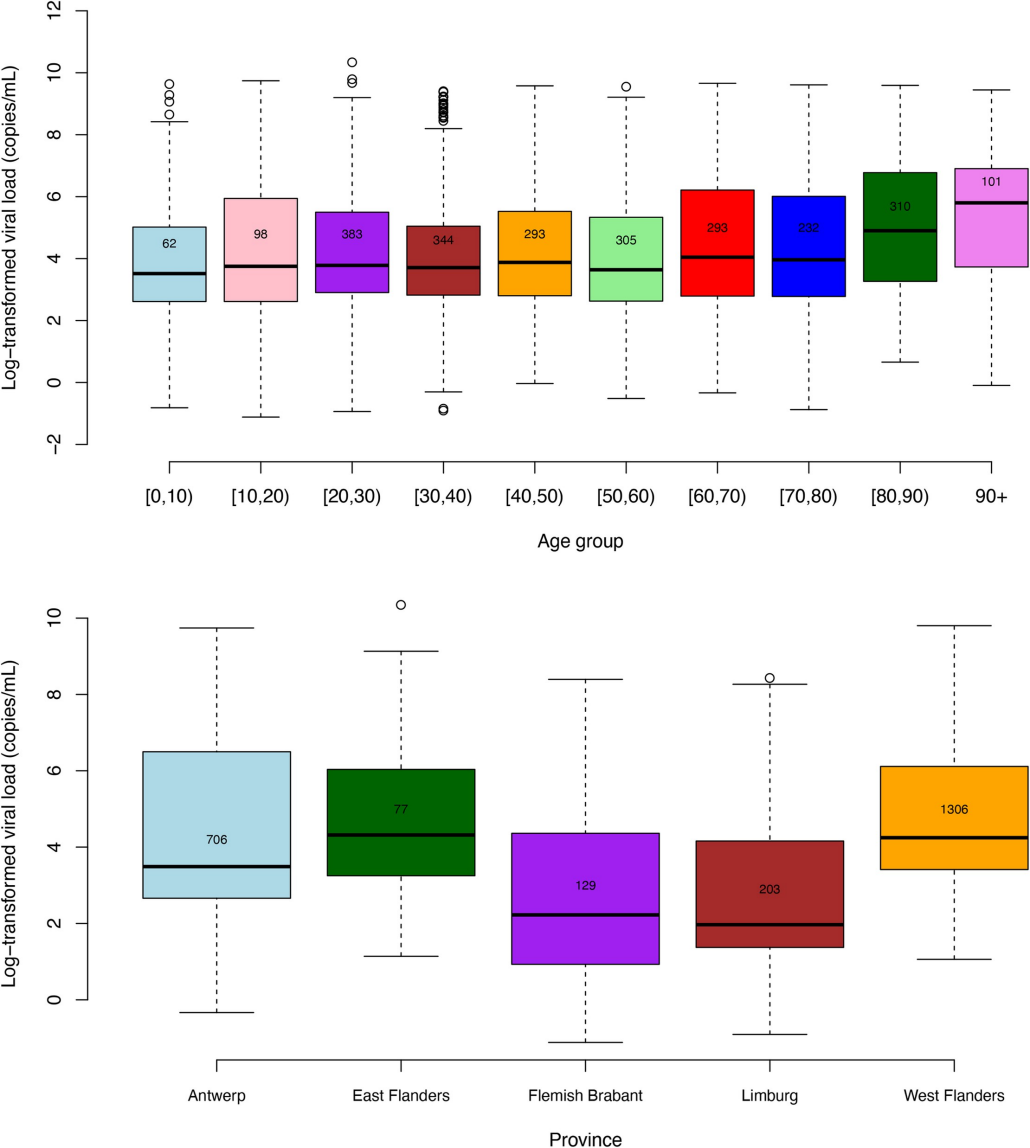
Boxplots of the log-transformed viral load (in copies/mL) for SARS-CoV-2 positive a-/pre-symptomatic patients by age (upper panel) and province (lower panel) with reported number of observations per group within the boxplots and data collected between 1 April 2020 to 31 March 2021.

The distributions of the log-transformed viral load by age (A) and province (B) are depicted in Fig 4. The evolution of the proportion of strongly positive individuals by age and province are included in S2A and S2B Fig.

The overall Kruskal-Wallis two-sided p-value to test for differences in median viral load among age groups was found to be smaller than 0.0001. In general, patients in the oldest age categories ([80, 90) and 90+ years of age) were found to have the highest viral loads, which were not significantly different (p-value = 0.268) among themselves, while having pairwise BH corrected p-values all being smaller than 0.010. Similar to age, significant differences in median viral load were observed across provinces (two-sided p-value < 0.0001) overall, and between West Flanders and all other provinces, except for East Flanders (p-value = 0.725), based on the pairwise comparisons. The median viral loads in Limburg and Flemish Brabant were not significantly different (p-value = 0.484). The pairwise difference in median viral loads between Antwerp and East Flanders is borderline non-significant (p-value = 0.052).

**Fig 5:**
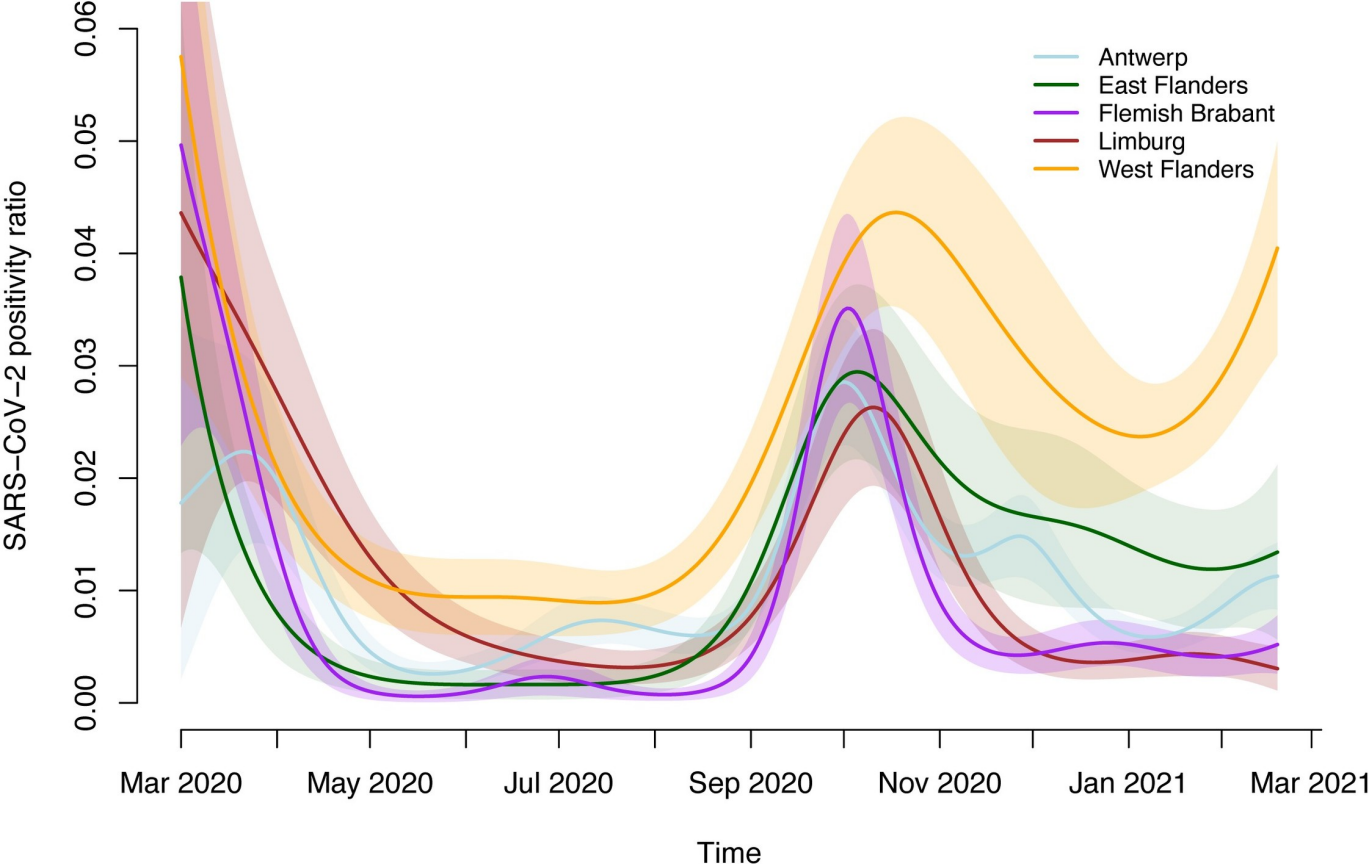
Estimated evolution of the SARS-CoV-2 real-time RT-PCR positivity ratio in a-/pre-symptomatic patients screened pre-hospitalization/pre-surgery by province for individuals aged 54 years (median age of screened patients) together with pointwise 95% confidence bounds (shaded areas).

**Fig 6:**
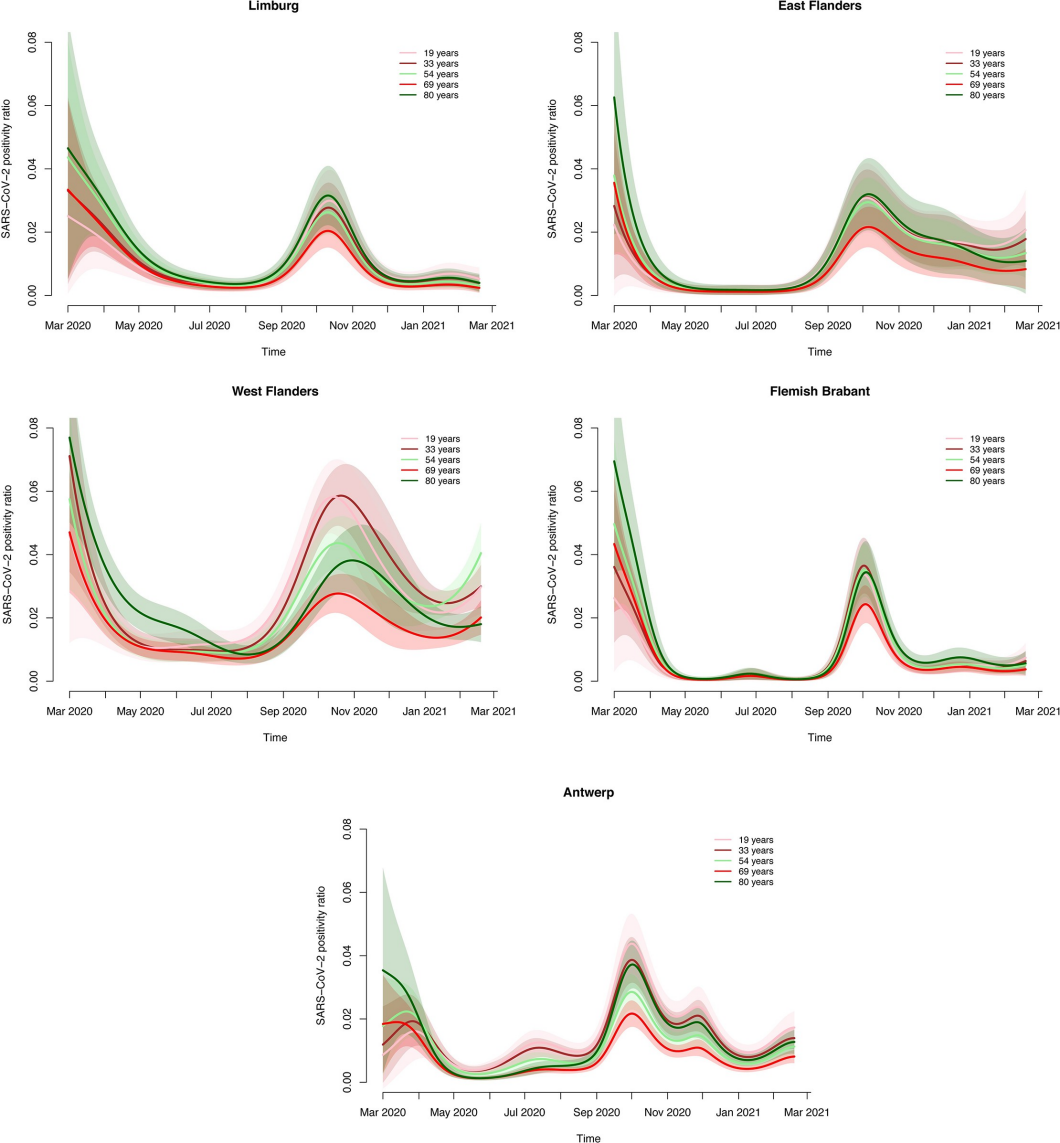
Estimated evolution of the SARS-CoV-2 real-time RT-PCR positivity ratio in a-/pre-symptomatic patients screened pre-hospitalization/pre-surgery by age (individuals aged 19 years, 33 years, 54 years, 69 years and 80 years) in Limburg (upper left panel, A), East Flanders (upper right panel, B), West Flanders (middle left panel, C), Flemish Brabant (middle right panel, D), Antwerp (lower panel, E) together with pointwise 95% confidence bounds (shaded areas).

The final GAMs to model the SARS-CoV-2 positivity ratio and the mean log-transformed viral load included a fixed effect of province, and smooth functions of age and time, with the later time evolutions being province-specific. In order to show the effect of age, time and province on the SARS-CoV-2 positivity ratios we constructed different figures. Fig 5 depicts the estimated evolution of the SARS-CoV-2 positivity ratio by province over time for patients of age 54 years (median age of all screened patients). Clearly, the estimated positivity ratio is highest in West Flanders during the second wave for patients aged 54 years. In general, the peak in SARS-CoV-2 positivity is observed to be between the beginning and mid October 2020 with some delay in West Flanders (end of October), at least for this age cohort. In general, the positivity ratio remains relatively high during the inter-wave period between May 2020 and September 2020. A similar observation is made for other age cohorts as displayed in Fig 6. More specifically, trends in estimated positivity ratio are different between provinces, but similar between age cohorts (except for West Flanders). The evolution of the positivity ratio is shown for patients of age 19 (10% percentile), 33 (first quartile), 54 (median age), 69 (third quartile) and 80 years of age 90% percentile). In general, 69 year old patients have the lowest positivity ratio during the second COVID-19 wave. SARS-CoV-2 positivity is high among 19 year old during the second wave which is dissimilar to the situation during the first COVID-19 wave.

Similar plots for the log-transformed viral load can be found in S3 and S4 Figs. On average, the median viral load reaches the highest levels in West Flanders. Although there is no clear temporal pattern in the mean log-transformed viral loads across all Flemish provinces, an increase is observed in median viral load between July 2020 and September 2020, for patients of all ages, in Limburg and Flemish Brabant, prior to their increases in positivity ratio and the start of the second COVID-19 wave in Belgium. In general, there are no substantial age differences in terms of the median viral load, and the evolution thereof is very similar for each age within a province. Note that the number of data points is limited in this analysis, except in West Flanders, which is reflected in the uncertainty around the estimated mean values.

## Discussion

Surveillance of (the evolution of) the fraction of asymptomatic COVID-19 cases in healthcare settings can be an important tool to avoid and reduce nosocomial infections since a-/pre-symptomatic patients potentially have high viral loads [27]. In addition, pre-admission and pre-surgery screenings in hospitals provide additional information concerning SARS-CoV-2 carriership in the population of a-/pre-symptomatic SARS-CoV-2 infected individuals. To our knowledge, this is the first study investigating the SARS-CoV-2 positivity ratio and viral load in asymptomatic patients screened pre-hospitalization or pre-surgery.

We report high positivity ratios of SARS-CoV-2 (overall 1.27%) amongst asymptomatic patients screened pre-hospitalization or pre-surgery in one of the nine included hospitals located in the Flemish provinces of Antwerp, East Flanders, West Flanders, Limburg and Flemish Brabant throughout the investigated period (1 April 2020 to 31 March 2021). The highest peak % SARS-CoV-2 carriership was observed during the first and second Belgian COVID-19 wave (i.e., 3.59% in April and 3.00% in November 2020) [28]. The highest positivity ratios were observed in West-Flanders with high case numbers observed in the beginning of 2021 which can be explained by outspoken endemic circulation of the South African 501Y.V2 SARS-CoV-2 variant in the general population, also referred to as the beta variant or B.1.351. Lowest positivity ratios were observed in July 2020 after a period of a full lockdown, implementation of non-pharmaceutical interventions and only minor easing of containment measures (e.g., social distancing, increased hygiene, face-and-mouth masks,…). The evolution of the positivity ratio in our study population resembles the evolution in hospitalizations and positivity ratio in the general Flemish population. However, the SARS-CoV-2 peak occurred earlier for the latter (October instead of November 2020), which may be explained by the fact that in October the testing strategy was changed and the focus was put on screening symptomatic patients leading to a higher positivity ratio in the general population. Also the median viral load and proportion of strongly positive patients follow a similar temporal evolution as was seen in the positivity ratio. While a decrease in viral load is observed after the first wave, median values started to increase from 3 months before the second wave until the end of the second wave. Our results agree with the findings of Dobrovolny *et al*., stating that relaxing of social distancing measures too quickly could lead to a rapid rise in the number of cases, driven in part by asymptomatic infections which remain largely undetected in the absence of large-scale testing and tracing efforts [29]. The hypothesis of underestimated viral circulation in Belgium based on confirmed cases only has also been put forward by Herzog *et al*. based on the analysis of serial serological data [30].

Young COVID-19 patients are known to be mainly asymptomatic or pauci-symptomatic [7,31]. Children may act as hidden drivers of the pandemic, although schools were found not to be a high risk setting for transmission of SARS-CoV-2 during the first and second wave of infections and several studies found that school closures did not contribute to the control of the epidemic [32,33]. In Belgium, the second SARS-CoV-2 wave started after reopening of the schools, however, we observed a real-time RT-PCR SARS-CoV-2 positivity ratio of 0.93% in children (0–18 year old) and no higher SARS-CoV-2 positivity ratio in children compared to adults. Our results agree with the study of Ingelbeen *et al*. who showed that the proportion of cases among 10–19-year-olds in Brussels did not significantly change after school reopening [34]. If children were equally susceptible, one might expect even higher ratios of carriership, since they were less restricted in the number of contacts (i.e., due to attendance of summer camps, and given that schools were open as of 1 September 2020 onward). On the other hand, it is likely that a substantial part of the children requiring hospitalization or surgery during the epidemic period (when non-urgent care was often scaled down given the high pressure of COVID-19 on regular care) had fewer social contacts due to serious or chronic illness. Furthermore, only a small number of children was included in the study.

Our results show significantly higher SARS-CoV-2 ratios in 80+ year old asymptomatic patients compared to younger patients across all time periods, except for the summer months. This may reflect the increased transmission risk in residential care homes and/or an increased transmission of SARS-CoV-2 in the non-nursing home 80+ population, especially during the first wave [35]. The lower positivity ratio in the summer months may be due to stricter measures for residents of retirement homes compared to the younger population. While the positivity ratio during the first wave was highest for the 80+ age group, a significantly different positivity ratio was observed for both the 80+ age group as well as the 20–40 age group compared to the other age groups during the second wave. Moreover, the highest positivity percentage was observed in 20–30 year old individuals in August-September 2020 before the second COVID-19 wave emerged (potentially attributable to returning travelers), indicating that this age group has been largely affected during the second COVID-19 wave and individuals therein could be viewed as important drivers of that wave. In general, 69 year old patients had the lowest positivity ratio during the second COVID-19 wave, which may be explained by the fact that they had no potential work-related exposure and no risk factor of residing in a nursing home.

Some studies report no significant differences in SARS-CoV-2 viral load in respiratory samples received from different age groups [8,36,37]. In contrast, Salvatore *et al*. observed that SARS-CoV-2 viral loads were significantly higher among participants under 18 years of age [38]. Children with COVID-19 are reported to have moderate or high viral loads regardless of age, symptoms or severity of infection [39]. More specifically, children younger than 5 years with mild to moderate COVID-19 have been reported to have high amounts of SARS-CoV-2 viral RNA in their nasopharynx compared with older children and adults [40]. However, children with asymptomatic SARS-CoV-2 infection had lower levels of virus in the nasopharynx/oropharynx than symptomatic children, but timing of infection relative to diagnosis likely impacted levels in asymptomatic children [6]. In our study, we observed the highest viral loads in asymptomatic 80+ year old individuals. The combination of higher SARS-CoV-2 positivity ratios and higher viral loads in this age group can be explained by frequent outbreaks seen in retirement homes [35,41].

The main limitation in this study includes the underrepresentation of the younger and overrepresentation of the elder age groups (with co-morbidities requiring hospitalization or surgery) within the study cohort. Secondly, (weak positive) asymptomatic cases could have corresponded to previous infections with persistent existence of detectable RNA, although this limitation does not affect the main findings of this study. Further, pre-hospitalization and pre-surgery screening policies may have been different between the participating hospitals and over time and the use of different RT-PCR platforms complicates the interpretation of viral loads. In addition, the initial RT-PCR was not repeated when testing negative and was only performed on upper respiratory tract samples, no virus genome sequencing was performed which could have yielded additional information and no information regarding the development of symptoms later on was available. Although clustering of observations within hospitals is not accounted for in the GAM models for the SARS-CoV-2 positivity ratio, these models do include provincial effects (i.e., reflecting spatial differences in disease incidence during the course of the epidemic), thereby covering the spatiotemporal evolution of the SARS-CoV-2 transmission dynamics. Consequently, the previously mentioned GAMs assume that observations within individual hospitals are independent. Alternatively, one could consider a generalized additive mixed model (GAMM) including hospital as a random effect, however, this did not result in a better fit to the observed data implying limited within-hospital association between observations. In general, these flexible additive models show similarly different evolutions of the SARS-CoV-2 positivity ratio across Flemish provinces, induced by a different timing and extent of local transmission after seeding of new SARS-CoV-2 cases during the summer period prior to the second COVID-19 wave in Belgium.

To conclude, SARS-CoV-2 highly circulated in all age groups (overall 1.27%) among asymptomatic patients with strong spatiotemporal differences and a predominance and highest viral loads observed in the elderly (80+ year old) except between the first and second COVID-19 wave. Moreover, ahead of the second COVID-19 wave an increase in median viral load was noted with the highest overall positivity ratio observed in 20–30 year old individuals, indicating they could have been the hidden drivers of this wave.

## Supporting information

S1 Fig Evolution of the bi-weekly SARS-CoV-2 real-time RT-PCR positivity ratio in a-/pre-symptomatic patients screened pre-hospitalization/pre-surgery by Flemish province as a function of time (from 1 April 2020 to 31 March 2021). The size of the dots is proportional to the number of observations that are available.
(TIF)

S2 Fig A. Evolution of the proportion of strongly positive patients among the PCR positive patients screened pre-hospitalization/pre-surgery by age group. B. Evolution of the proportion of strongly positive patients among the PCR positive patients screened pre-hospitalization/pre-surgery by Flemish province.
(TIF)

S3 Fig Evolution of the mean log-transformed viral load in positive patients screened pre-hospitalization/pre-surgery by Flemish province as a function of time (from 1 April 2020 to 31 March 2021) with pointwise 95% confidence bounds (shaded areas). (TIF)

S4 Fig Evolution of the mean log-transformed viral load in positive patients screened pre-hospitalization/pre-surgery by age (individuals aged 19 years, 33 years, 54 years, 69 years and 80 years) in Limburg (upper left panel, A), East Flanders (upper right panel, B), West Flanders (middle left panel, C), Flemish Brabant (middle right panel, D), Antwerp (lower panel, E) together with pointwise 95% confidence bounds (shaded areas).
(TIF)
