# Supplementary figures and images for: Evaluation of the SARS-CoV-2 positivity ratio and upper respiratory tract viral load among asymptomatic individuals screened before hospitalization or surgery in Flanders, Belgium
Source: PLoS One. 2021 Nov 11;16(11):e0259908. doi: 10.1371/journal.pone.0259908 (PMC8584712; doi:10.1371/journal.pone.0259908)

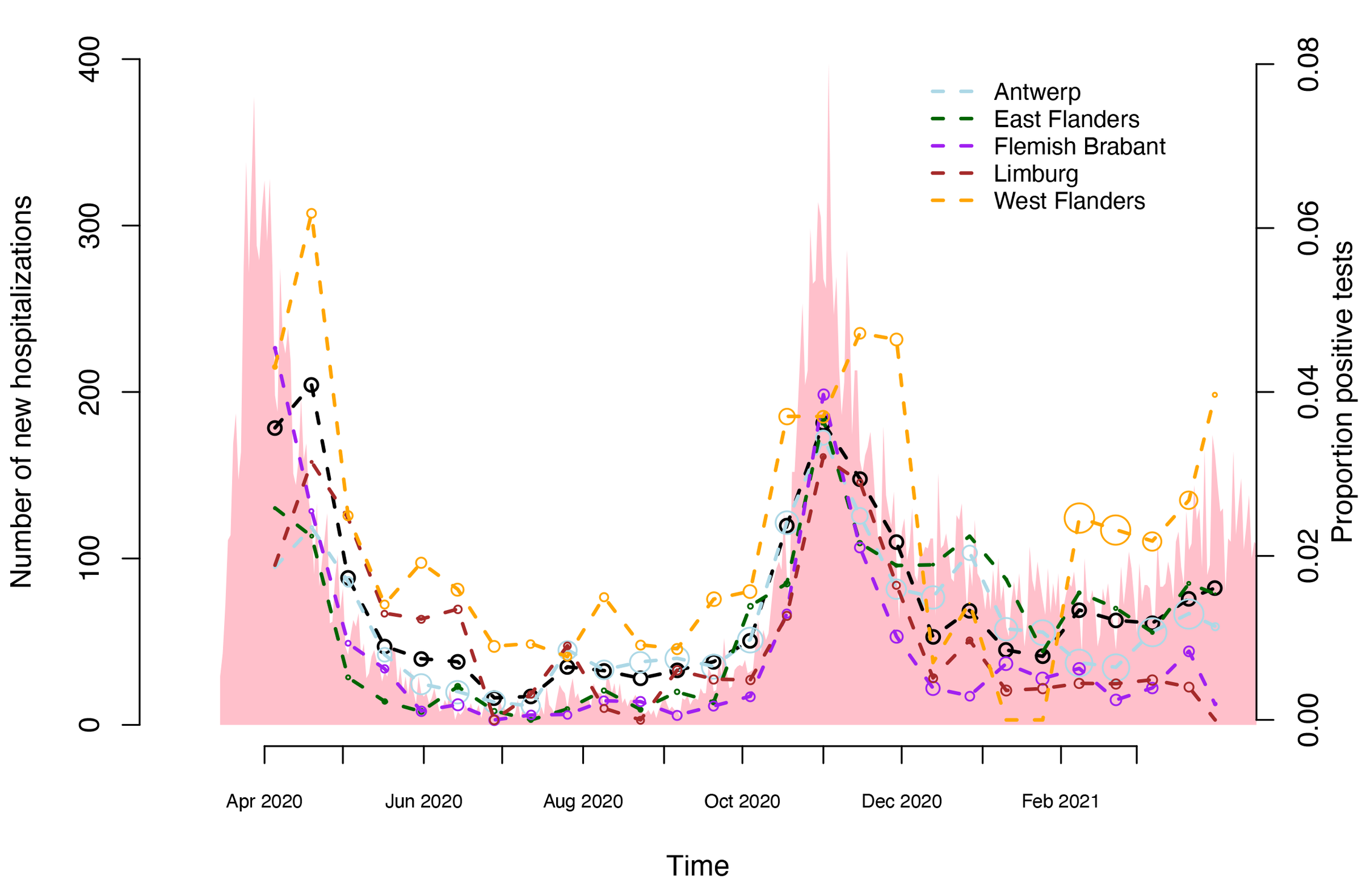

Supplement: S1 Fig — The size of the dots is proportional to the number of observations that are available. (TIF) [file pone.0259908.s001.tif]

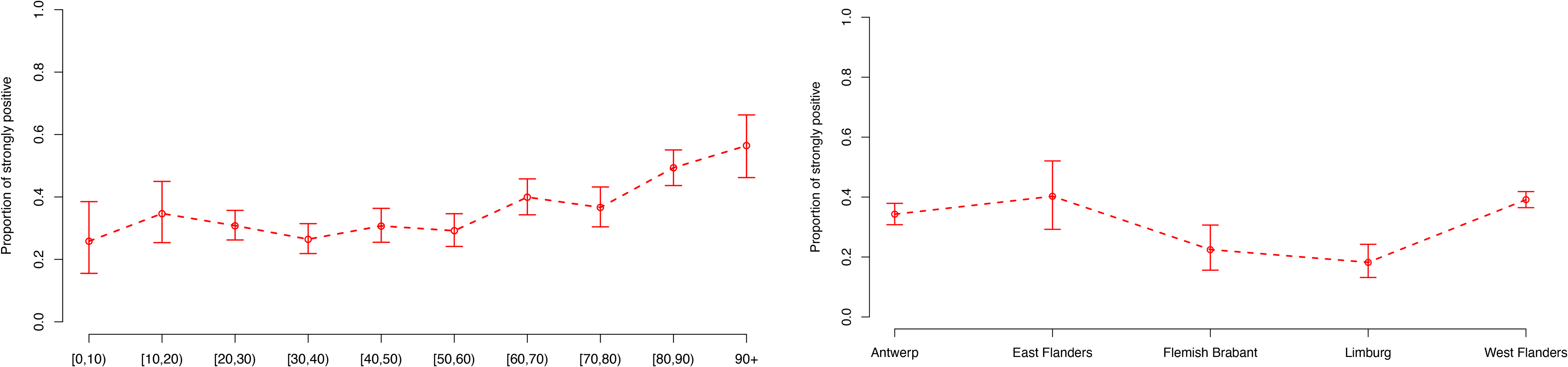

Supplement: S2 Fig — A. Evolution of the proportion of strongly positive patients among the PCR positive patients screened pre-hospitalization/pre-surgery by age group. B. Evolution of the proportion of strongly positive patients among the PCR positive patients screened pre-hospitalization/pre-surgery by Flemish province. (TIF) [file pone.0259908.s002.tif]

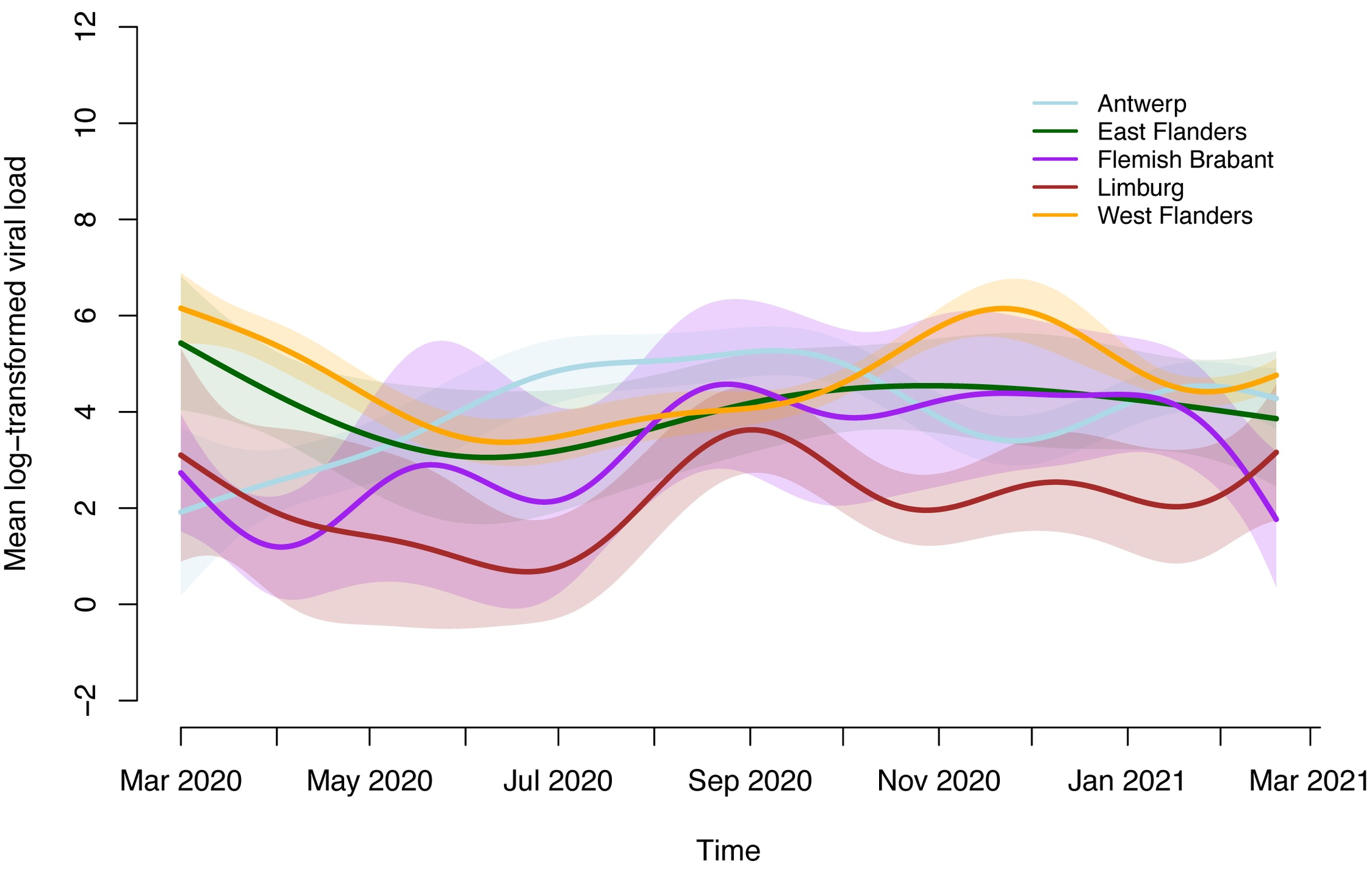

Supplement: S3 Fig — (TIF) [file pone.0259908.s003.tif]

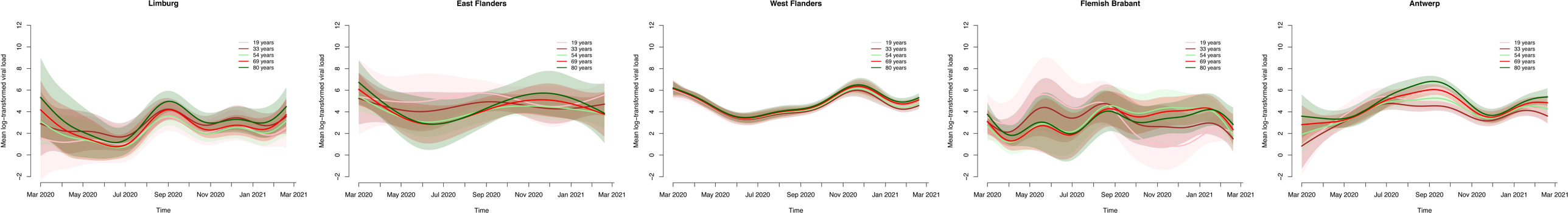

Supplement: S4 Fig — Evolution of the mean log-transformed viral load in positive patients screened pre-hospitalization/pre-surgery by age (individuals aged 19 years, 33 years, 54 years, 69 years and 80 years) in Limburg (upper left panel, A), East Flanders (upper right panel, B), West Flanders (middle left panel, C), Flemish Brabant (middle right panel, D), Antwerp (lower panel, E) together with pointwise 95% confidence bounds (shaded areas). (TIF) [file pone.0259908.s004.tif]
